# Developing an Innovative System of Open and Flexible, Patient-Family-Centered, Virtual Visiting in ICU During the COVID-19 Pandemic: A Collaboration of Staff, Patients, Families, and Technology Companies

**DOI:** 10.1177/08850666211030845

**Published:** 2021-10

**Authors:** Kathleen A. S. Thomas, Bernadine F. O’Brien, Agatha T. Fryday, Ellen C. Robinson, Marissa J. L. Hales, Sofia Karipidis, Aaron Chadwick, Kimberley J. Fleming, Alan P. Davey-Quinn

**Affiliations:** 1 Department of Intensive Care, College of Intensive Care Medicine of Australia and New Zealand, Illawarra Shoalhaven Local Health District, Wollongong Hospital, Wollongong, NSW, Australia; 2 Department of Intensive Care, Illawarra Shoalhaven Local Health District, Wollongong Hospital, Wollongong, NSW, Australia

**Keywords:** virtual visiting, video conferencing, open and flexible visiting, tailored system of virtual visiting, HowRU, intensive care unit, COVID-19, visitor restrictions, patient-family-centered care, patient family communication, patient-family connect

## Abstract

Few challenges of the COVID-19 pandemic strike at the very core of our humanity as the inability of family to sit at the bedside of their loved ones when battling for their lives in the ICU. Virtual visiting is one tool to help deal with this challenge. When introducing virtual visiting into our ICU, we identified 5 criteria for a sustainable system that aligned with patient-family-centered care: virtual visiting needed to (1) simulate open and flexible visiting; (2) be able to accommodate differences in family size, dynamics, and cultural practices; (3) utilize a video conferencing platform that is private and secure; (4) be easy to use and not require special teams to facilitate meetings; and (5) not increase the workload of ICU staff. There is a growing body of literature demonstrating a global movement toward virtual visiting in ICU, however there are no publications that describe a system which meet all 5 of our criteria. Importantly, there are no papers describing systems of virtual visiting which mimic open and flexible family presence at the bedside. We were unable to find any off-the-shelf video conferencing platforms that met all our criteria. To come up with a solution, a multidisciplinary team of ICU staff partnered with healthcare technology adoption consultants and two technology companies to develop an innovative system called HowRU. HowRU uses the video conferencing platform Webex with the integration of some newly designed software that automates many of the laborious and complex processes. HowRU is a cloud based, supported, and simplified system that closely simulates open and flexible visiting while ensuring patient and family privacy, dignity, and security. We have demonstrated the transferability of HowRU by implanting it into a second ICU. HowRU is now commercially available internationally. We hope HowRU will improve patient-family-centered care in ICU.

## Introduction

Of the many challenges that the COVID-19 pandemic has brought, few strike at the very core of our humanity as the inability of family to sit at the bedside of their loved ones when battling for their life in the Intensive Care Unit (ICU). We have seen this distressing impact of visitor restrictions documented in both pubic media^1-3^ and medical literature alike.^4-7^ Not only are patients and their families affected, but ICU staff too. Moral injury can occur when ICU staff are unable to facilitate holistic care, which involves providing emotional support and guidance for the families throughout their ICU journey, particularly at the end of a patient’s life.^6^ A recent study looking at the mental wellbeing of intensive care staff across 21 French ICUs during the COVID-19 pandemic found that regret about visiting restriction policies was an important determinant of poor mental health.^7^

It is no surprise, then, that in today’s era of modern technology there has been an almost intuitive move to virtual visiting, using video conferencing to bridge the physical gap between patients and families across the world. Virtual visiting has been endorsed by organizations such as the Australia and New Zealand Society of Intensive Care,^8^ Society of Critical Care,^9^ Intensive Care Society,^10^ and the International COVID-19 Intensive Care Advisory Group.^11^ In a recently published national survey of ICUs across the UK, 97% had some form of virtual visiting established.^12^ When understanding the challenging conditions under which many ICUs have been running during the pandemic, the ability to quickly implement virtual visiting is remarkable.

Across the literature we see extensive heterogeneity in how virtual visiting is facilitated. The most obvious variation is the use of different video conferencing platforms: in the UK, for example, at least 19 different video conferencing platforms were used across the NHS.^12^ Many other components of virtual visiting vary too. We see variation in the type and set up of device used for virtual visiting (from mobile devices held by staff or mounted to stands, to existing bedside computers with mounted cameras, to existing telecritical care systems), the number and availability of devices used within units (from one device per patient, to one or a small handful of devices shared across the unit or hospital), and the manner in which virtual visiting is coordinated and facilitated (from a communication team often consisting of additional staff, to a re-purposed telecritical care coordination center, to bedside nurses).^13-23^

While there have been limited formal qualitative studies, the feedback about virtual visiting from patients, families, and staff is grossly positive. Patients are sent love and emotional support and are reminded of what waits for them when they get better.^13^ This provides patients with a sense of hope and motivation to engage in therapies, thereby promoting physical recovery.^12^ Virtual visiting is also perceived as therapeutic in being able to help re-orientate patients with delirium, and overcoming language/communication barriers.^12^ Families appreciate receiving information and emotional support.^12^ They are reassured and alleviated of a sense of helplessness. They value feeling connected to their loved ones, witnessing their loved ones being well cared for and treated as a person, and sharing stories of a patient’s legacy with healthcare staff.^13,14,16,21,23^ Staff have described the experience of virtual visiting as “surprisingly intimate and rewarding,” finding that it fosters a deeper connection with and recognition of the person in the bed^14^; other staff report it being a “profoundly powerful experience.”^13^ In the National UK Study, 68% perceived virtual visiting as improving staff morale.^12^

Taken together, the current literature clearly shows immense value in having some sort of system of virtual visiting in the ICU when physical visitation is restricted. The next questions that arise are: is there an optimal system of virtual visiting? What have we learned from the heterogeneous methods trialed thus far? How can virtual visiting best align with patient-family-centered care?

Sasangohar et al, who re-purposed their existing telecritical care system for virtual visiting, performed a qualitative evaluation of 59 participants and offer valuable insight into what families suggest in order to improve virtual visiting. Over half of the participants expressed the desire to have on-demand access to the technology to initiate calls instead of the limited access controlled by the coordination center. Other areas of improvement included improved scheduling processes and improved technical capabilities.^16^

Another study looking at family and staff perspectives of telephone and video communication in ICU during the pandemic found that the main suggestion to improve virtual visiting was to use technology that more closely approximated the experience of families being at the bedside. Specifically, these suggestions included positioning the camera so that the family can see the patient and their surroundings, offering families the opportunity to ask questions about tubes and devices, and offering time for patients and families to interact without clinician participation.^24^

While these areas of improvement pertain to the particular model of virtual visiting used by Sasangohar et al and Kennedy et al, the suggestions for improvements made by families are likely broadly applicable to many of the other published systems that demonstrate similar limitations in terms of restricted access to video calls, utilizing platforms not specifically designed for virtual visiting in the ICU setting, and laborious organization of each virtual visit. Families from Sasangohar et al and Kennedy et al’s studies are essentially asking us to offer what would be considered the usual standard of practice in many ICUs—open and flexible visiting—but to do this in a virtual format. We are now starting to see this request echoed professionally, by colleagues suggesting that the concept of ‘open visitation’ could be further broadened to include “virtual open visitation.”^25^

We know of the constellation of adverse phycological outcomes that can occur in ICU patients and their families, termed ICU trauma and post-intensive care syndrome-family respectively.^26-31^ We also know that providing open and flexible visiting has the effect of reducing the risk of developing such outcomes.^32-34^ While having some sort of virtual visiting may reduce this risk of developing ICU trauma and post-intensive care syndrome-family, having on-demand virtual access for patients and families that meets their needs for information and support is more likely to further reduce the risk.^35^

The authors of the National UK study state that “although family members might prefer on-demand access to virtual visiting, workload and privacy concerns make this prohibitive.”^12^ We, however, disagree. In this paper, we will present a new system of open and virtual visiting, HowRU, that does meet families’ wishes for on-demand access, while ensuring patient privacy, dignity, and data security. HowRU is a cloud-based, fully supported, easy-to-use system that requires no technical expertise and places minimal additional workload on ICU staff.

In this article we examine the barriers we encountered to using off-the-shelf video conferencing products in the ICU setting for virtual visiting. We then describe the criteria we devised for a functional and sustainable system of virtual visiting that simulates our usual standard of open and flexible visiting. This is followed by a discussion of the unique collaboration between multidisciplinary ICU staff, patients, families, a technology adoption specialist company, and 2 technology companies to devise our tailored solution, HowRU, to meet these criteria. We then describe how HowRU works in our unit, and the various stages of development, culminating in the implantation of HowRU into another ICU. Lastly, we discuss the potential challenges others may encounter when implementing HowRU into a new ICU.

## Why Off-the-Shelf Video Conferencing Platforms Failed to Meet the Needs of Our Intensive Care Unit

When trying to quickly implement virtual visiting at the beginning of the COVID-19 pandemic, we explored many off-the-shelf video conferencing platforms. We expected this to be a straightforward endeavor, but found this not to be the case. Here we discuss the barriers we encountered to using off-the-shelf video conferencing products for virtual visiting in our ICU.

### Using Patient’s Private Accounts

When thinking about our unconscious or incapacitated ICU patients, it was immediately apparent that it was not appropriate to use patients’ private accounts such as Facebook, WhatsApp or Facetime. This would require obtaining patients’ private account details and ICU staff signing in on the patient’s behalf. This was a clear breach of patient privacy and therefore precluded its use in our ICU.

### Using Generic Accounts for the ICU

The alternative to using patients’ private accounts was to create generic accounts for the ICU using off-the-shelf video conferencing platforms. These generic accounts could then be shared between patients. There were several barriers that arose when attempting this solution.

#### Accounts requiring a phone number

On platforms where a phone number is used to make video calls, there is a risk of inadvertently giving families a contact number that is presumed to belong to the ICU. This has led to situations where subsequent calls from family members have caused distress to both parties.^20^ There is also the risk of families being able to call into these generic accounts when other families are using the account, risking breeches in patient confidentiality.

The only way to avoid establishing an inappropriate 2 way line of communication using shared accounts would be for staff to always block the caller details, meaning all video calls would have to be outbound and scheduled. This creates the problem with scheduling discussed in the next section.

#### Accounts using scheduled virtual meetings

Many video-conferencing platforms create scheduled virtual meetings. An invitation link, meeting ID, or passcode can then be sent out to family members by email or text message. While this ensures a secure meeting space, the major issue is the process through which these meetings are scheduled and facilitated. Scheduling, and then communicating the meeting details to family members, is a multistep, time-consuming process that requires coordination across the unit to equitably share time and to ensure meetings will not overlap.

Having to send email or text message invites to virtual meetings also raises the question of how to maintain a digitally secure list of family contacts easily accessible to the clinical team in ICU. ICU staff will need to locate, confirm, and re-enter phone numbers or email addresses each time a video call is made. This is another step that adds to the workload of overstretched clinical staff.

As we see in the literature, some units have been able to overcome the issues with scheduling and facilitating calls by having a dedicated team whose job it is to organize virtual meetings with families. These teams have consisted of nurse practitioners, medical students, residents, non-ICU seconded medical staff, or pre-existing telecritical care coordination centers. We did not have the option of developing a separate virtual visiting coordination team; the virtual visits would have to be facilitated by our existing clinical staff, most likely the bedside nurses. Not only would it be unfair to place an additional workload on our clinical staff, we also knew that having to use new technology would be stressful and challenging, if not impossible, for some of our clinical staff. We felt confident that as a unit in the short term we could pull together to overcome these challenges, like has been done in many units globally, but it was clear that this was not a sustainable long-term solution.

### Scheduled Virtual Meetings Did Not Adhere to Our Usual Standard of Open and Flexible Family Visiting

We identified that having limited scheduled meetings did not align with our usual practice of open and flexible family visiting. Having to schedule meetings did not allow the flexibility that is often required in the dynamic ICU environment, and does not accommodate for the common scenario of family members being in different time zones. Scheduled meetings do not provide families the opportunity to check in on their loved ones when they feel the need to, leaving them feeling disempowered and anxiously awaiting the next scheduled meeting. We saw this first hand expressed by the families of our first COVID-19 patients when trialing scheduled virtual visiting using off-the-shelf video conferencing platforms.

We also recognized the importance of not limiting the number of family members that could be involved in a virtual meeting. We wanted to be able to accommodate the differences in family size, preferred type of interactions, complex dynamics, and cultural practices. We wanted to ensure that family members were not excluded, and that no single family member was burdened with the sole responsibility of communicating often complex and confronting medical information to other family members.

### Barriers When Trying to Simulate Open and Flexible Visiting

To provide more open and flexible virtual visiting, we sourced an iPad for each ICU bed. We then explored having a private and secure account for each patient where we could create a virtual meeting space for the family that could accommodate multiple family members if required. The barrier that we came up against was how to create a secure account for each patient easily. All the video conferencing platforms required an accessible unique email address to create and then verify a new account using 2-step verification. There was no simple or straightforward way of establishing an email account for each patient, nor for going through the process of opening and verifying each new account.

## Our Criteria for a Functional and Sustainable System of Virtual Visiting That Simulates Open and Flexible Visiting

At this point we realized off-the-shelf video conferencing solutions were unable to meet the needs of our ICU. The limitations of applying a platform designed for 2 non-disabled and conscious people to the unique constraints of our ICU setting were clear.

We summarized the criteria for a functional and sustainable system of virtual visiting for our ICU as the following. Virtual visiting should:

1. Utilize a video conferencing platform that is private and secure in line with hospital data protection guidance
2. Closely simulate our usual standard of open and flexible visiting
3. Be flexible to accommodate the differences in our patients’ family size, preferred type of interactions, complex dynamics, and cultural practices
4. Be easy to use, require minimal technical skills, and require no special teams to organize or facilitate meetings
5. Not increase the workload of the ICU staff

## Finding a Solution

### Collaboration

In coming up with a solution to address the criteria listed above, with the support of our hospital executive and IT department, we partnered with healthcare technology adoption consultants from the company Taleka, who then collaborated with the technology companies Cisco and Citrus Health. Our ICU working party consisted of doctors, nurses, social workers, and administration staff, as well as former ICU COVID-19 patient families.

### The Solution: HowRU

The solution we came up with is HowRU. HowRU is a fully supported, cloud-based system of virtual visiting that utilizes the video conferencing platform Webex, with the addition of innovative automated programming to tailor to our ICU needs.

**Figure 1.**
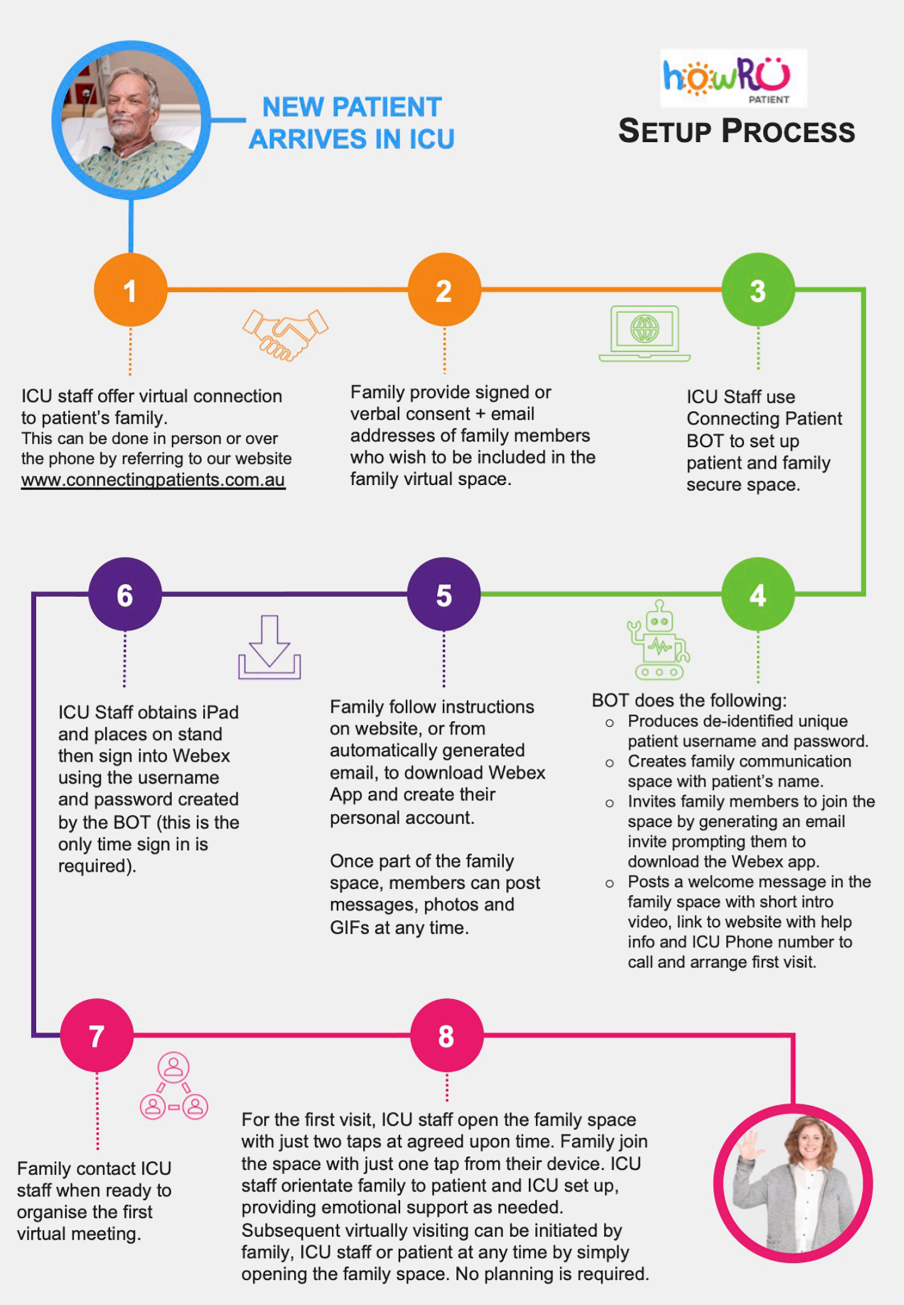
Workflow from new patient arriving in ICU to virtual family visitation.

The workflow from the time a patient arrives in ICU to virtual visiting with family is demonstrated in Figure 1 and is described below.

#### How HowRU works

When a new patient arrives in ICU we offer the patient and/or family access to virtual visiting through HowRU. We refer the family to our website at www.connectingpatients.com.au where they can find all the information they need to understand and use HowRU. This information is presented in instructional videos, guides, and links to download Webex on different devices, with a clear layout of the steps to initiate the first virtual visit and troubleshooting information. The website also contains a consent form that covers the rules of use, which families must agree to prior to their first virtual visit. Having all this information on an accessible website precludes the need to physically hand out information and is essential when visitors are unable to enter ICU or family members are not local.

**Figure 2.**
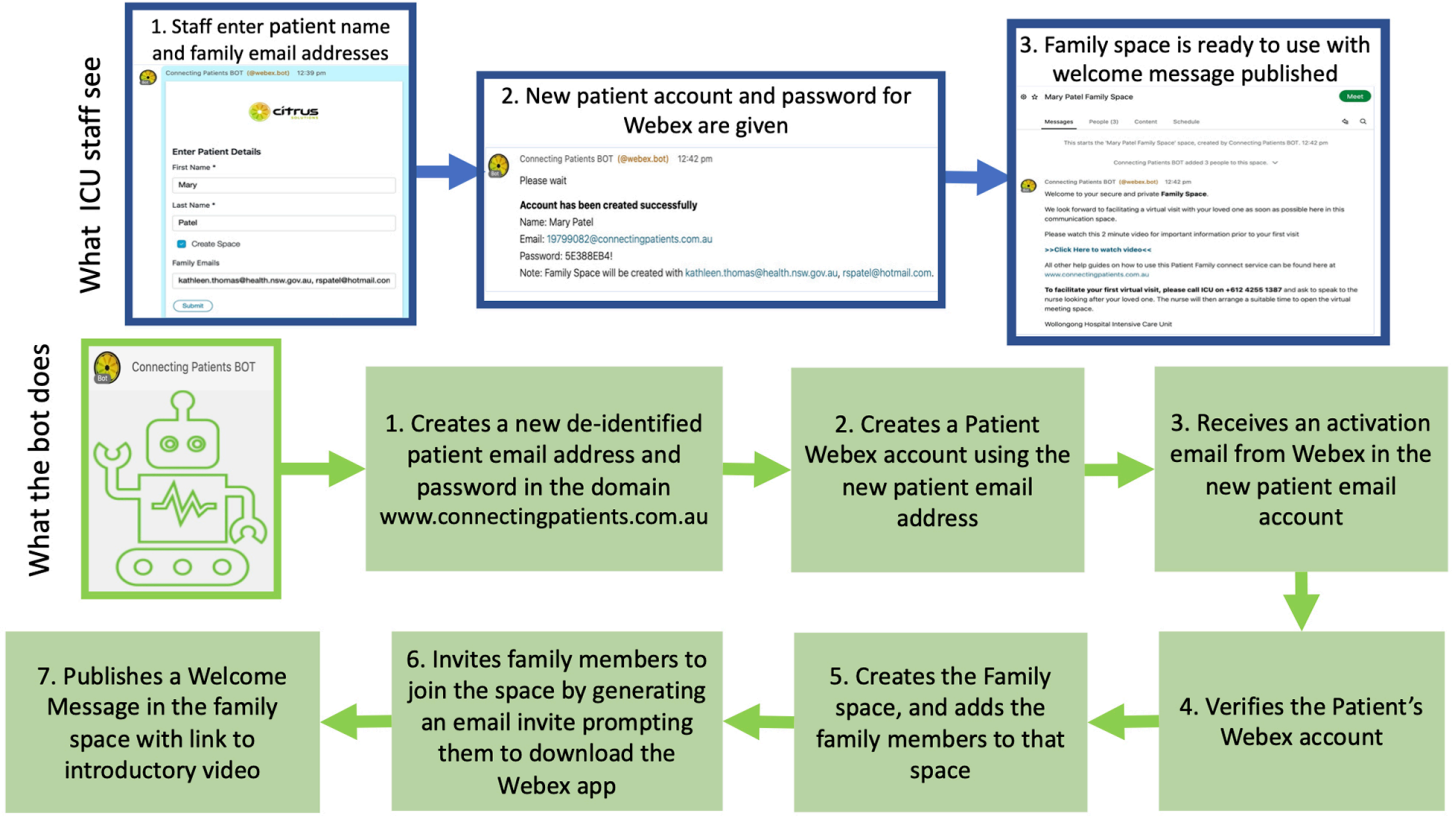
The automation that simplifies HowRU: What the staff see while the bot automatically completes 7 steps to set up a patient account, create a family virtual meeting space, invite family to join the virtual meeting space and publishe a welcome message.

After consent is obtained, the ICU staff set up a patient account using an account creation bot (an automated software program designed to perform a specific task) on one of our central computers. As seen in Figure 2, the staff simply enter the patient’s name and the email addresses of family members wanting to virtually visit, and the bot performs multiple tasks to produce and verify a new Webex patient account. The bot produces a unique de-identified username and password for the patient’s account. The bot also creates a virtual meeting space for the family inside Webex, invites family members to join the family space by triggering off email invites, and publishes a welcome message in the family space that includes a link to an introductory video as well as a reminder on how to initiate the first virtual visit.

**Figure 3.**
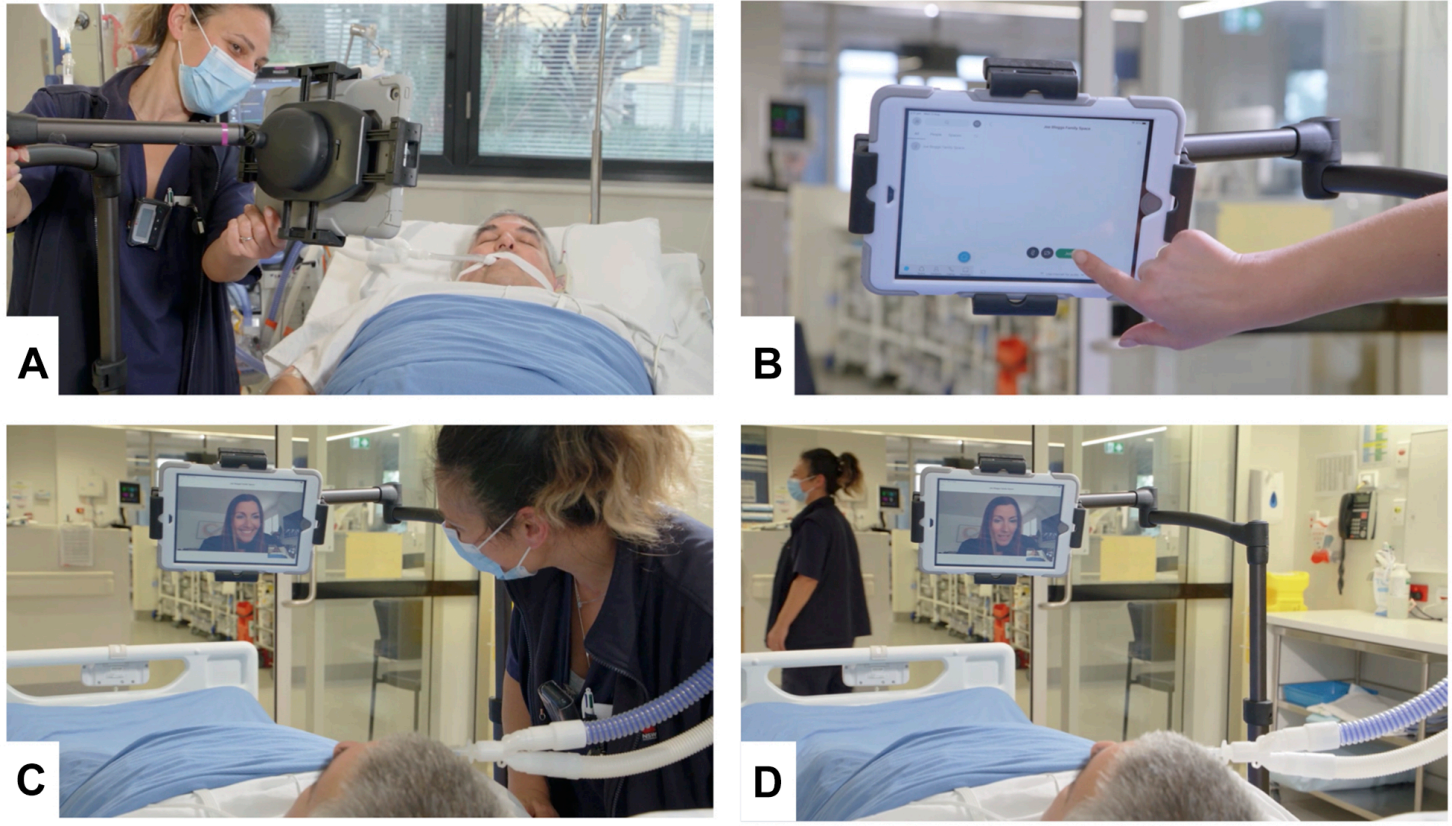
Set up of HowRU in the patient bay in ICU.   A, Bedside nurse places the iPad with HowRU ready to go on the adjustable stand at the bedside. B, Bedside nurse opens the family virtual meeting space with 2 taps. C, Bedside nurse interacts with the family member in the virtual family meeting space. D, Bedside nurse leaves the family member to spend time alone with the patient. *Note*: This is a simulated set up.

This bot is a key component of HowRU. It takes away from our clinical staff all the time-consuming and complex IT tasks required to set up and verify a private and secure patient account that abides by our hospital data protection guidance.

Our staff then obtain an iPad assigned to the patient and sign into Webex using the username and password produced by the bot. This is a one-time sign in and no further security steps are required. The staff place the iPad in a wheeled adjustable stand, positioned appropriately for the patient (Figure 3a).

Once the family have downloaded the Webex app to their individual devices, the first virtual meeting is set up. This is a facilitated meeting where ICU staff prepare the family prior to directing the video onto the patient, ensuring the families are supported when confronted with seeing their loved one in ICU for the first time. The bedside nurse opens the family virtual meeting space with just 2 taps on the iPad (Figure 3b). Each family member receives a notification on their device telling them the family virtual meeting space is open for them to visit, which they can do with just 2 taps on their personal device. Once the family is oriented and comfortable, the staff can then leave the bedside with the family virtual meeting space open so that family members can come and go as they please (Figure 3c and 3d).

After that first visit, the family space can be opened easily at any time by the bedside nurse. This can occur at the discretion of the bedside nurse whenever there is time available during the day, at a formally organized time, or, if appropriate, at the spontaneous request of a family member. Family members can visit individually or as a group. There is no limit on how many family members can be in the virtual meeting space at any one time.

This simulates our usual practice of flexible and open visiting, accommodating families of all sizes and in different time zones. It also means that when a family member wakes up in the middle of the night worrying about their loved one, they can simply ask the bedside nurse to open the family space so they can virtually check in and feel connected to their loved one. Family members can have unrushed time visiting their loved one together or separately and can at different times interact with the bedside nurse, or be left alone with their loved one in privacy. Should the nurse be required to attend to the patient privately while the virtual room is open, they simply leave the space with one tap, and can re-open the space when finished with their task.

When the patient leaves the ICU, the patient’s account is deleted. The family will still have access to all the messages in the family space from their personal devices, but the patient’s account is deleted and therefore no personal data can be accessed from the ICU iPad.

#### HowRU facilitates families to support each other in non-COVID-19 patients

While initially designed with COVID-19 patients in mind, as hospital visiting and travel restrictions limit those able to be present the bedside in non-COVID-19 patients, we have found HowRU important not just for virtual visiting, but also as a means for families to support each other. One example is an elderly man whose children all lived overseas, attending his critically unwell wife in ICU. He was alone and lacked confidence in his ability to understand medical information. When he would visit his wife, or attend family meetings, his children would use HowRU to be present with him virtually. This allowed the children to participate in their mother’s care journey and support their father with shared decision making.

#### Other benefits of HowRU

Other benefits of HowRU include:

- Multimodal communication is possible: photos, text messages, voice messages and GIFs can be sent between patients and families at any time allowing a range of options for each individual to best express themselves. This is particularly useful for patients who are conscious but unable to speak, such as those with a tracheostomy.
- Much like an ICU diary, the families have a record of their interactions with their loved one throughout their journey in ICU which can be helpful in processing their ICU experience.
- The system is adaptable for each stage of the patient’s journey through ICU. As the patient becomes more independent, they can take on a more active role in managing their interactions in the family virtual meeting space. The same is true in reverse if the patient were to deteriorate.
- There is enormous potential for additional ways in which HowRU could be used in ICU. For example, we have integrated our hospital chaplain service into HowRU. If a patient requires a chaplain visit, the chaplain can do this virtually either individually or with family members present. Other potential uses would include communication between patients and health care workers, inter-departmental communication, consults and supervision.

## The Key Pieces That Make HowRU a Tailored and Supported System, Not Just an App

There are 3 key components that make HowRU a sustainable system. The first key component is the automated patient account creation and family space creation using the innovative bot as discussed above. The second component is the support for families, which has also been discussed above. The third component is the support for ICU staff.

We support our ICU staff in the following ways:

- **HowRU Champions**: We have trained 12 social work, nursing, and administration champions in our unit to use HowRU in its entirety. The training takes approximately one hour. The champions are then able to train other ICU staff as they go, assist with common issues that arise, and escalate issues they cannot fix. The champions are also in a Webex space with experts from Taleka and Citrus Health where they can ask questions to learn collectively. These champions receive quarterly refresher training.
- **Step-by-step guide**: We have designed and tested a “step-by-step guide” that takes staff through the entire process, from obtaining the iPad to starting a virtual meeting with the family. It also includes basic troubleshooting.
- **Video**: We have developed an instructional video to complement the step-by-step guide.
- **Help Line:** A help line has been established by our hospital IT and Citrus Health to provide support via phone for issues that arise.
- **Simple workflow design:** We have developed a streamlined workflow for the unit, which is clearly outlined in our flowsheet.

## Development, Testing, and Ongoing Improvements of HowRU

**Figure 4.**
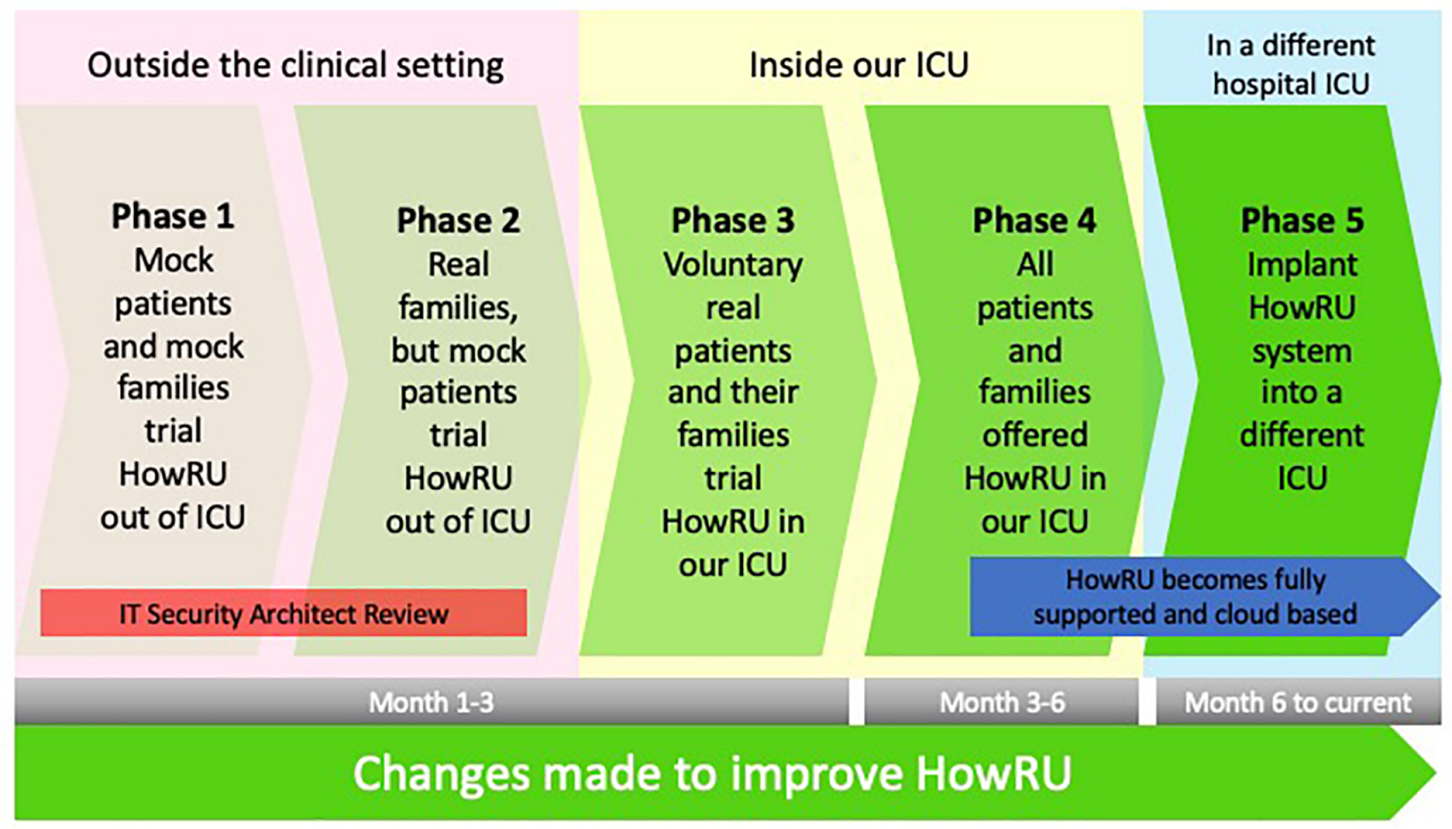
Testing and development process of HowRU.

The process of developing HowRU has occurred over 5 phases as demonstrated in Figure 4. The entire process has been collaborative between our ICU working party, technology adoptions specialists from Taleka, and technology experts from our hospital IT department, Citrus Health and Cisco. Technology adoption specialists from Taleka were vital to this process, as they acted as intermediaries and translators between our ICU team and the technology companies. Our ICU working party presented to Taleka the problems we encountered with off-the-shelf video conferencing platforms and explained the criteria we needed to have a functional virtual visiting system. Taleka then worked with the technology experts to develop a cohesive solution that met our needs.

At each phase of development, we have taken feedback from those using the system, including the patients, their families, and ICU staff to improve upon HowRU.

In phases 1 and 2, we trialed the prototype, ironed out all the early issues, and developed a lot of the support material. We also formally consulted family members of former COVID-19 ICU patients for their input into system design and usability. An important piece of this early development was the review and subsequent modifications suggested by our IT Security Architect to ensure appropriate hospital-level data security. This guidance aligns with that produced by The Intensive Care Society’s Legal and Ethical Advisory Group.^10^

In phase 3 we introduced HowRU into our ICU and sought out patients and families who were willing to trial virtual visiting and provide us with feedback. Once we were confident that HowRU was fully functional, we began phase 4 where we were able to offer HowRU to all our ICU patients and families.

During phase 4, HowRU was transformed into a fully managed and supported cloud-based service. This was an important transition for several reasons: (1) It provided an agile service that was able to adapt to the everchanging technology, ensuring the website, videos, guides, and champions were all kept up to date; (2) It reassured us that if there was an issue with connectivity, particularly during a critical moment for a family, there was help available; (3) It allowed HowRU to be easily implanted into another unit. This led to phase 5, where HowRU was introduced into a different hospital ICU. That implementation was very successful and was ready for live patients and families within a timeframe of 3 weeks once all the existing support content was tailored and the iPads had been configured. Less than 24 hours after training was completed for the Champions, a live patient-family connection occurred.

HowRU is now well established in 2 ICUs and is being reviewed and trialed by others. We continue to take feedback from all users to improve HowRU. It has been designed to evolve as new technology emerges and is easily adapted for improvements.

## Potential Challenges in Implementing HowRU Into a New ICU

When introducing HowRU into a new ICU, there are several challenges that may arise. We believe the biggest challenge will be getting key decision-makers to understand and value “the why”: why families and patients need open and flexible virtual visiting; why off-the-shelf video conferencing platforms are not adequate; why having a secure and protected virtual space is essential; and why a supported system is important for staff and families. Communicating and justifying “the why” may be particularly difficult in units where there is no pre-COVID culture of open and flexible visiting. It may also be difficult in units that have come up with some sort of family-patient-staff communication solution during COVID. In such units, the level of expectation and what is considered sufficient, even if sub-optimal, may already be set and difficult to re-imagine.

From a monetary perspective, there are potential challenges in justifying the return on investment of HowRU because the returns of are not easily quantifiable. The more obvious benefits around patient-family-centered care, mental health of patients and families, and staff morale have been discussed in this article. There are additional benefits, however, such as improved patient-family communication and overall satisfaction, which could reduce complaints and the cost associated with addressing these. In addition, many hospitals have relied upon teams staffed by medical students, healthcare workers seconded from other departments, or volunteers to organize communication in ICUs during the pandemic. As a result, the true cost of a communication team may be hidden. This makes it difficult to compare existing communication costs with HowRU. In addition, video is seemingly so available in the world that it is commonly viewed as being a free commodity. Those without insights into the unique challenges of the ICU may struggle to appreciate the need to pay for a unique service.

The maximal benefit of HowRU comes from patients that are unconscious or dependent on ICU staff for communication. Therefore, in lower acuity ICUs where there are more patients able to use their own devices, the perception of the value of HowRU may be diminished.

Once “the why” is clear and accepted, “the how” will be much easier. However, like the successful implementation of any new product, the uptake of HowRU will depend on strong leadership, buy-in from staff, local champions, effective messaging, appropriate training, incentivization, and the development of policy and systems to standardize its use. One major advantage of HowRU is that the work required for implementation is in large part done by the technology adoption specialists who support the ICU through the implementation process. The technology adoption specialists provide training, develop messaging material, liaise between IT and clinical staff, and problem-solve technical and non-technical issues that arise. This significantly reduces the ICU investment in time, energy, and creativity. It also greatly reduces the burden and stress that many healthcare workers experience when adopting new technology.

## Conclusion

There is no equal substitute for being able to offer physical comfort to a loved one who is critically ill or dying. However, when visitation in person is not possible, we can now provide open and flexible virtual visiting that aligns with patient-family-centered care using HowRU. HowRU aligns with our usual code of conduct, ensuring patient and family privacy, dignity, and security. It facilitates an open and flexible line of communication that can be adapted to the needs of each individual patient and family. HowRU is a tailored and supported system that is simple to use, requires no special technical expertise, and places minimal additional workload upon ICU staff. We have demonstrated that HowRU can easily be implanted into another ICU.

It has been predicted that the psychological impact of COVID 19-related separation on ICU families will reverberate for years, and likely result in high numbers of people needing trauma-related services.^36^ We sincerely hope that our system of open virtual visiting will minimize the harmful effects of visitor restriction during such critical and often life-changing moments in our patients’ and families’ lives.

HowRU is now commercially available internationally in any country with Cisco Webex access. More information can be found at https://www.citrushealth.com.au/ and https://www.taleka.com/howru/.
